# Effect of Degradation on the Physicochemical and Mechanical Properties of Extruded Films of Poly(lactic acid) and Chitosan

**DOI:** 10.1021/acsomega.3c09296

**Published:** 2024-02-13

**Authors:** José Ramón Flores-León, Dora Evelia Rodríguez-Félix, Jesús Manuel Quiroz-Castillo, Heidy Burrola-Núñez, María Mónica Castillo-Ortega, José Carmelo Encinas-Encinas, Juana Alvarado-Ibarra, Hisila Santacruz-Ortega, Jesús Leobardo Valenzuela-García, Pedro Jesús Herrera-Franco

**Affiliations:** † Departamento de Investigación en Polímeros y Materiales, Universidad de Sonora, C.P. 83000 Hermosillo, Sonora, Mexico; ‡ Licenciatura de Ecología, Universidad Estatal de Sonora, C.P. 83100 Hermosillo, Sonora, Mexico; § Departamento de Ingeniería Química y Metalurgia, Universidad de Sonora, C.P. 83000 Hermosillo, Sonora, Mexico; ∥ Centro de Investigación Científica de Yucatán, Unidad de Materiales, C.P. 97205 Mérida, Yucatán, Mexico

## Abstract

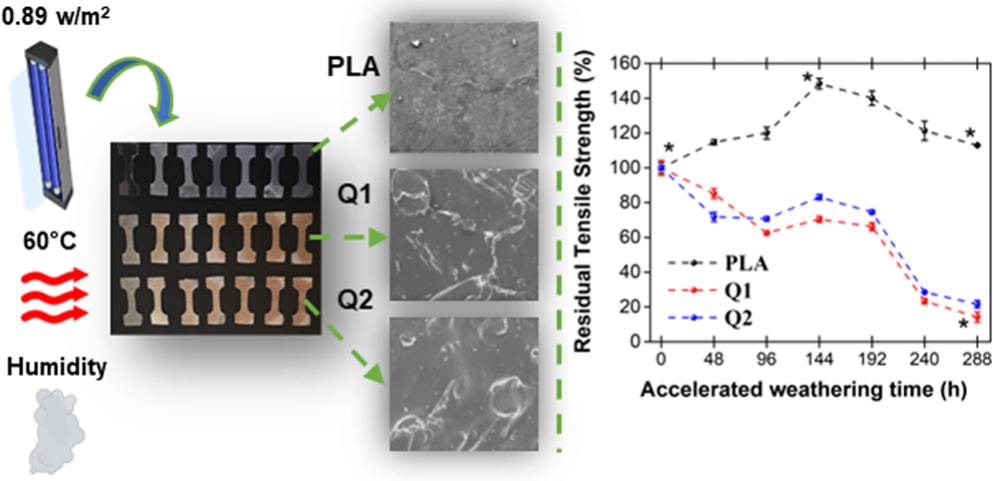

This study addresses the fabrication of extruded films using poly(lactic acid) (PLA) and chitosan, with and without maleic anhydride as a compatibilizing agent, for potential applications in disposable food packaging. These films underwent controlled conditions of UV irradiation, water condensation, and temperature variations in an accelerated weathering chamber. The investigation analyzed the effect of different exposure periods on the structural, morphological, mechanical, and thermal properties of the films. It was observed that PLA films exhibited a lower susceptibility to degradation compared to those containing chitosan. Specifically, the pure PLA film showed an increase in elastic modulus and strength during the initial 144 h of exposure, associated with cross-linking induced by UV radiation. On the other hand, film Q2 composed of PLA, chitosan, and maleic anhydride and Q1 without maleic anhydride experienced a tensile strength loss of over 50% after 244 h of exposure. The Q2 film exhibited greater homogeneity, leading to increased resistance to degradation compared to that of Q1. As the degradation time increased, both the Q1 and Q2 films demonstrated a decline in thermal stability. These films also exhibited alterations in crystallinity attributed to the chemo-crystallization process, along with fluctuations in the glass transition temperature and crystallization, particularly at 288 h.

## 1 Introduction

The escalating demand for plastic products, predominantly of synthetic origin, has led to concerning accumulation of waste in the environment. The production of food packaging and the widespread use of protective films in agriculture stand out as the primary contributors to this surge in plastic waste.^1−4^ Addressing the environmental repercussions of such waste necessitates the exploration of novel materials capable of degrading rapidly under ambient conditions, unlike the protracted degradation periods associated with conventional polymers.^5,6^

Although the integration of natural polymers and/or biopolymers into blends with synthetic polymers has been a common strategy to enhance properties and reduce degradation times, the 88% presence of synthetic polymers may pose environmental challenges during degradation. Therefore, the exclusive use of materials based solely on natural polymers or biopolymers becomes significant, offering the potential to reduce the environmental footprint.^7,8^

Poly(lactic acid) (PLA), a biodegradable and biocompatible polymer with mechanical properties akin to poly(ethylene terephthalate), emerges as a promising candidate for applications in disposable food packaging.^8^ Derived from renewable resources such as starch, PLA has a short shelf life and is compatible with food and beverages, making it ideal for applications like disposable packaging and agricultural mulch.^9−11^ On the other hand, chitosan is a cationic polysaccharide obtained through the deacetylation of chitin, the second most abundant natural polymer in nature.^12^ Chitosan, composed of 2-acetoamido-2-deoxy-d-glucopyrinose and 2-amino-2-deoxy-d-glucopyrinose linked by β (1 → 4) bonds, is characterized by being biodegradable and nontoxic to humans. Additionally, it possesses a high antimicrobial power attributed to the presence of the amino group in the glycosidic ring.^13,14^ This biopolymer exhibits susceptibility to different degrading agents, such as solar ultraviolet radiation, environmental humidity, and degradation through the action of microorganisms.^15^

Polymer degradation studies in nature are complex and time-consuming. Therefore, various alternatives have been sought to understand the physicochemical behavior of a material during its degradation but in shorter periods. In this regard, the use of accelerated degradation chambers is an excellent option to study the polymer degradation process. It allows simulating environmental conditions such as UV radiation, temperature, and humidity while intensifying them to obtain faster results.^15^ Research focused on studying the effect of chitosan on the degradation of composite materials has been reported. Huerta et al. (2015) explored the degradative behavior of PET/PLA and PET/chitosan films. Accelerated weathering tests indicated that the interaction between PET and chitosan favored the degradation rate compared to the PET/PLA mixture.^10^ Similarly, Lizárraga-Laborín et al. studied the degradative behavior of biodegradable films based on polyethylene, PLA, and chitosan. It was observed that the presence of chitosan favored the degradation of the material, with chitosan being the initial component that showed cracking during the degradation process.^16^

In this context, our study undertakes a comprehensive analysis of degradation-induced changes in extruded films of PLA and chitosan subjected to accelerated weathering conditions. We conducted a systematic study to assess the impact of different exposure periods on the physicochemical and mechanical properties of these biopolymer-based films. This research not only advances our understanding of degradation dynamics in such composite materials but also contributes to the development of sustainable alternatives with reduced degradation times, a critical advancement toward mitigating environmental concerns associated with plastic waste.

## 2 Experimental Section

### 2.1 Materials

Chitosan with a medium molecular weight (190,000–310,000 Da, 75–85% deacetylation) and maleic anhydride (MA) powder, 95% purity (molecular weight of 98.06 g/mol), were obtained from Sigma-Aldrich. Commercial PLA pellets (grade 2003d) with a molecular weight (Mw) of 120,000 Da, specified with a melt flow rate of 6.0 g/10 min (measured at 210 °C with a 2.16 kg load) and a density of 1.24 g/cm^3^, was sourced from NatureWorks. Chitosan and PLA were dried at 60 °C for 24 h before use, and MA was used as received.

### 2.2 Preparation of Extruded Films of PLA and Chitosan

To prepare the composite films, PLA pellets and chitosan were initially ground by using an MF 10 basic mill at 3000 rpm. PLA, chitosan, and MA were individually weighed, and mixtures were then prepared at different weight proportions, as specified in Table 1. Subsequently, the mixtures were homogenized by agitating them with a Booster electric mixer JJ-1 for 15 min through mechanical stirring. Following this, the polymer blends underwent further processing through extrusion molding. This was achieved using an Atlas laboratory mixer-extruder operating at 32 rpm and maintained at specific temperatures of 160 °C for the barrel and 170 °C for the dye.

**Table 1:** Concentration of Each Component in the Extruded Films

ID	PLA (wt %)	chitosan (wt %)	maleic anhydride (wt %)
PLA	100	0	0
Q1	90	10	0
Q2	89.75	10	0.25

### 2.3 Accelerated Degradation Studies

PLA, Q1, and Q2 films underwent accelerated degradation by using the Q-Lab QUV/se equipment, which includes UV exposure, water condensation, and irradiation control. Film degradation was analyzed at six different exposure time intervals (Table 2) following the conditions recommended by the ASTM 154 standard. This procedure involved two steps: (1) 8 h of UV irradiation using four 0.89 W/m^2^ lamps at 60 °C and (2) 4 h of water condensation at 40 °C.

**Table 2:** Exposure Time for the Accelerated Degradation of Films

ID	exposure time (h)
T0	0
T1	48
T2	96
T3	144
T4	192
T5	240
T6	288

### 2.4 Characterization

#### 2.4.1 Fourier Transform Infrared Spectroscopy (FTIR)

The films exposed to different degradation times were characterized by infrared spectroscopy to investigate the progression of degradation through the potential changes in the chemical structure of the polymers. This analysis was carried out using a Nicolet FTIR spectrophotometer in the ATR mode, over a frequency range from 4000 to 400 cm^–1^, with an average of 32 scans.

#### 2.4.2 Tensile Strength Test

The mechanical properties of the films were performed on a United SSTM-5kN universal machine equipped with a 5 kN load cell at a constant crosshead speed of 1 mm/min and a jaw spacing of 20 mm. The thickness of the films was determined with a Mitutoyo micrometer, and the dimensions were maintained in a range of 0.3 and 0.6 mm thickness and a width of 5.2 mm. An average of 10 specimens for each test is reported.

#### 2.4.3 Scanning Electron Microscopy (SEM)

The effect of the different degradation times on the film morphology was studied by SEM using a scanning electron microscope (JEOL JSM-5410LV) operating at 20 kV. The instrument was equipped with an INCA system and an energy-dispersive X-ray detector (Oxford Instrument). Samples were placed in copper sample holders using double-sided carbon tape and underwent a gold-plating process before analysis.

#### 2.4.4 Thermogravimetric Analysis (TGA)

The thermal stability of the films was determined by TGA using a PerkinElmer Pyris 1 TGA unit and a porcelain sample holder. Approximately 4 mg of each sample was weighed and heated within a temperature range from 27 to 600 °C at a heating rate of 10 °C/min in a nitrogen atmosphere. The residual material from the sample holder was then further heated to 900 °C at a rate of 50 °C/min under an oxygen atmosphere to eliminate any remaining residues.

#### 2.4.5 Differential Scanning Calorimetry (DSC)

The thermal properties of the films were studied through DSC using a PerkinElmer DSC 8500 analyzer. The initial heating was conducted at a rate of 10 °C/min, ranging from 0 to 200 °C, in a nitrogen atmosphere (flow rate of 20 mL/min). Following this, the samples underwent a cooling cycle and a second heating cycle under the same conditions as described above. The data obtained from the second heating were analyzed using the Pyris Manager Ink software.

## 3 Results and Discussion

### 3.1 Macroscopic Characteristics of Extruded PLA/Chitosan Films

**Figure 1:**
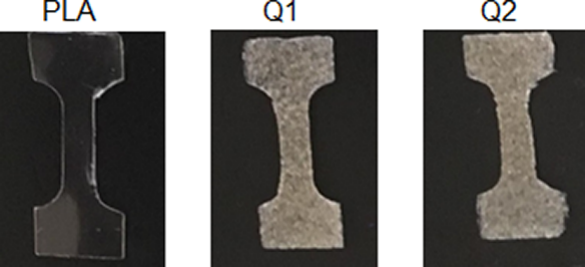
Digital images of the extruded films.

Extruded films were obtained using polymer mixtures of PLA/chitosan with and without MA, according to the compositions detailed in Table 1. The resulting films from pure PLA exhibited transparency, were colorless, and displayed homogeneity, while films from the polymeric mixture (Q1-Q2) presented a subtle yellow hue characteristic of chitosan particles, which were distributed in the PLA matrix (Figure 1). In general, the films obtained had dimensions of approximately 5.2 mm in width and 0.5 mm in thickness.

### 3.2 Accelerated Degradation Study

**Figure 2:**
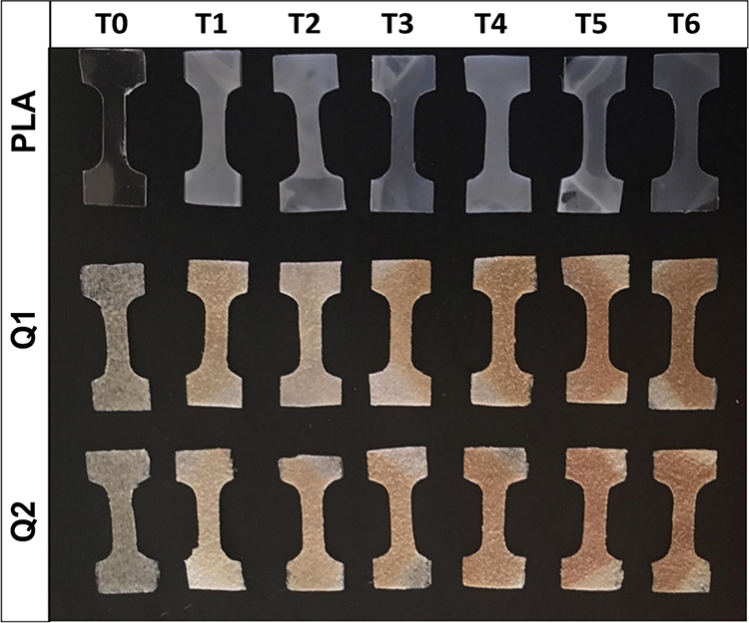
Digital images of the films corresponding to the different periods of exposure to accelerated degradation.

Figure 2 shows digital images of the extruded films exposed to different degradation times (T0–T6). As observed, the original PLA film (T0) presented a high degree of transparency due to its semicrystalline nature. However, with the extended exposure time, the film transitioned to an opaquer state, possibly due to an increase in crystallinity.^17,18^ This change persisted until the 288 h exposure mark. On the other hand, films Q1 and Q2 displayed notable alterations in color as the degradation time increased, undergoing a gradual shift from the initial yellow color to a darker orange hue. According to existing literature, these changes are related to the appearance of chromophore functional groups induced by radiation exposure.^19^ Additionally, isolated cracks were observed in the Q1 film after 240 h with a more pronounced manifestation during the final exposure time (T6). On the contrary, the Q2 film, which incorporates a compatibilizer, exhibited fewer and smaller cracks throughout the 288 h of degradation period, suggesting that the presence of MA confers some degree of resistance to degradation.

#### 3.2.1 Fourier Transform Infrared Analysis (FTIR)

**Figure 3:**
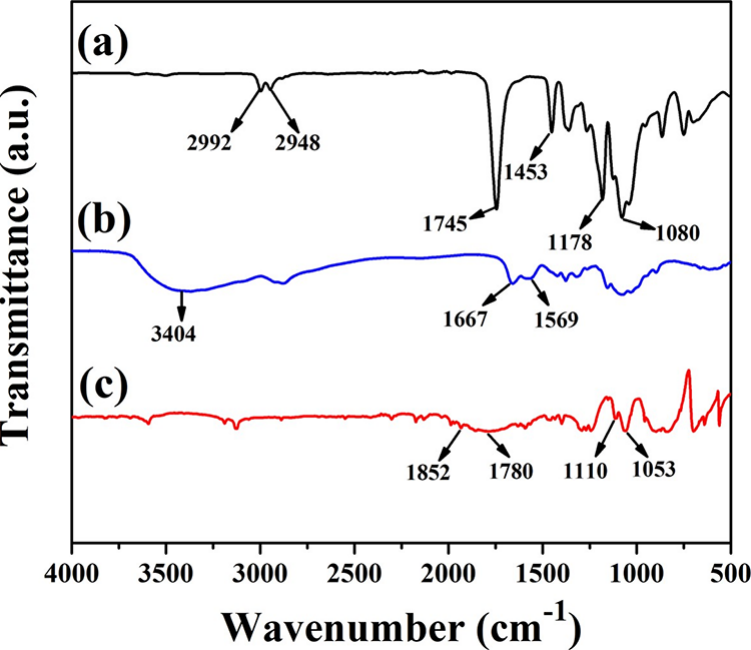
Infrared spectra of the pure components: (a) PLA, (b) chitosan, and (c) MA.

Figure 3 shows the infrared spectra for each of the pure components. PLA showed the characteristic signals of the ester functional group; in this way the stretching of the carbonyl group (C=O) appears at 1745 cm^–1^ and the asymmetric and symmetric stretching of the C–O–C bond at 1178 and 1080 cm^–1^, respectively. In addition, the signals corresponding to the asymmetric and symmetric stretching of the methyl group (−CH_3_) appear at 2992 and 2948 cm^–1^ and the asymmetric and symmetric bending of this group at 1453 and 1363 cm^–1^, respectively.^20,21^ The chitosan spectrum showed the characteristic signals of hydroxyl group stretching (−OH) at 3404 cm^–1^, amide-II (NH) bending at 1667 cm^–1^, and amine (NH_2_) twisting at 1569 cm ^–1^. On the other hand, the MA spectrum showed the signals attributed to the asymmetric and symmetric stretching of the carbonyl group at 1852 and 1780 cm^–1^, respectively, as well as the asymmetric and symmetric stretching of the C–O–C bonds at 1110 and 1053 cm^–1^, respectively.^22^

**Figure 4:**
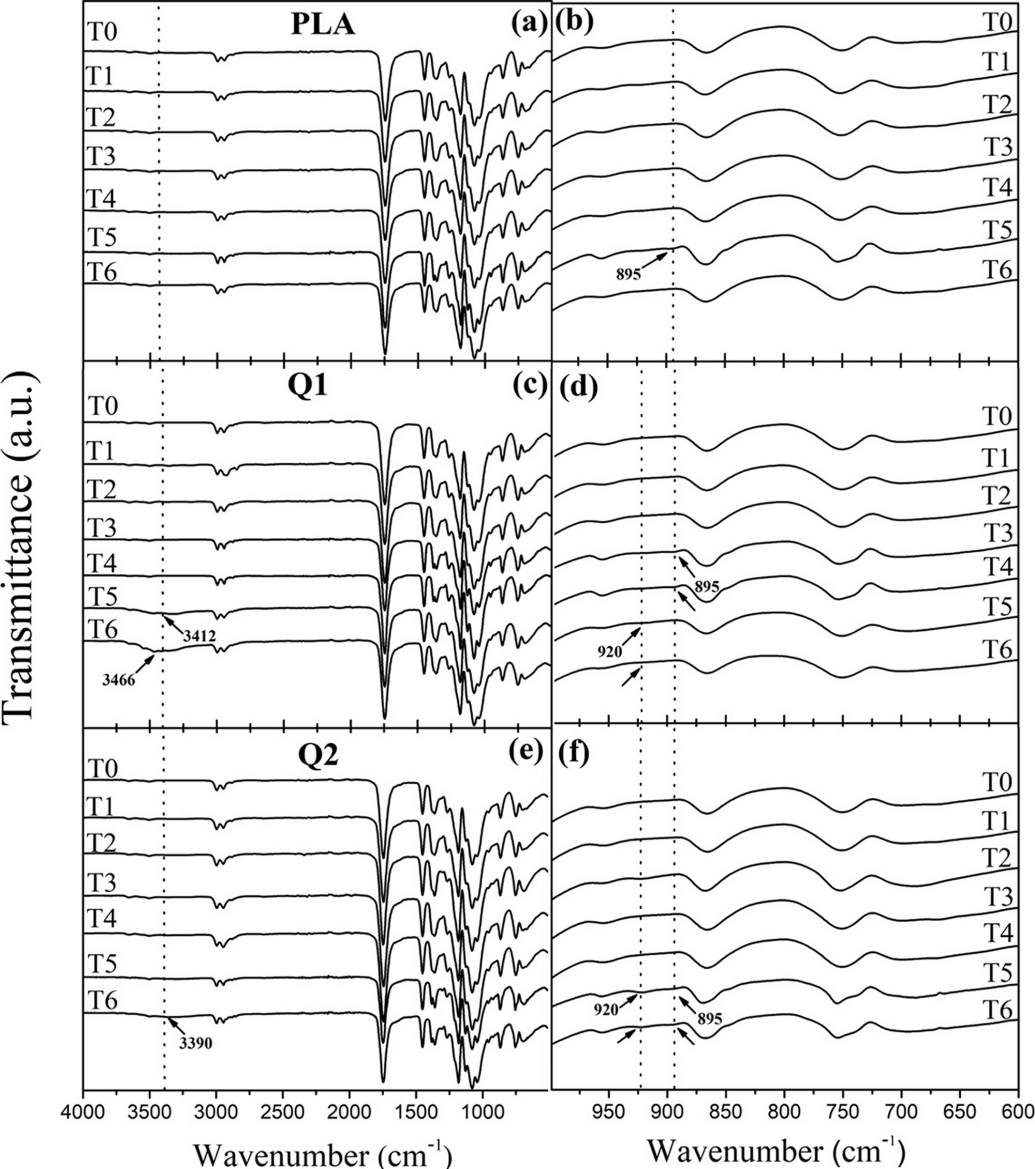
Infrared spectra of (a,b) PLA, (c,d) Q1, and (e,f) Q2 films at different exposure times of accelerated weathering.

**Figure 5:**
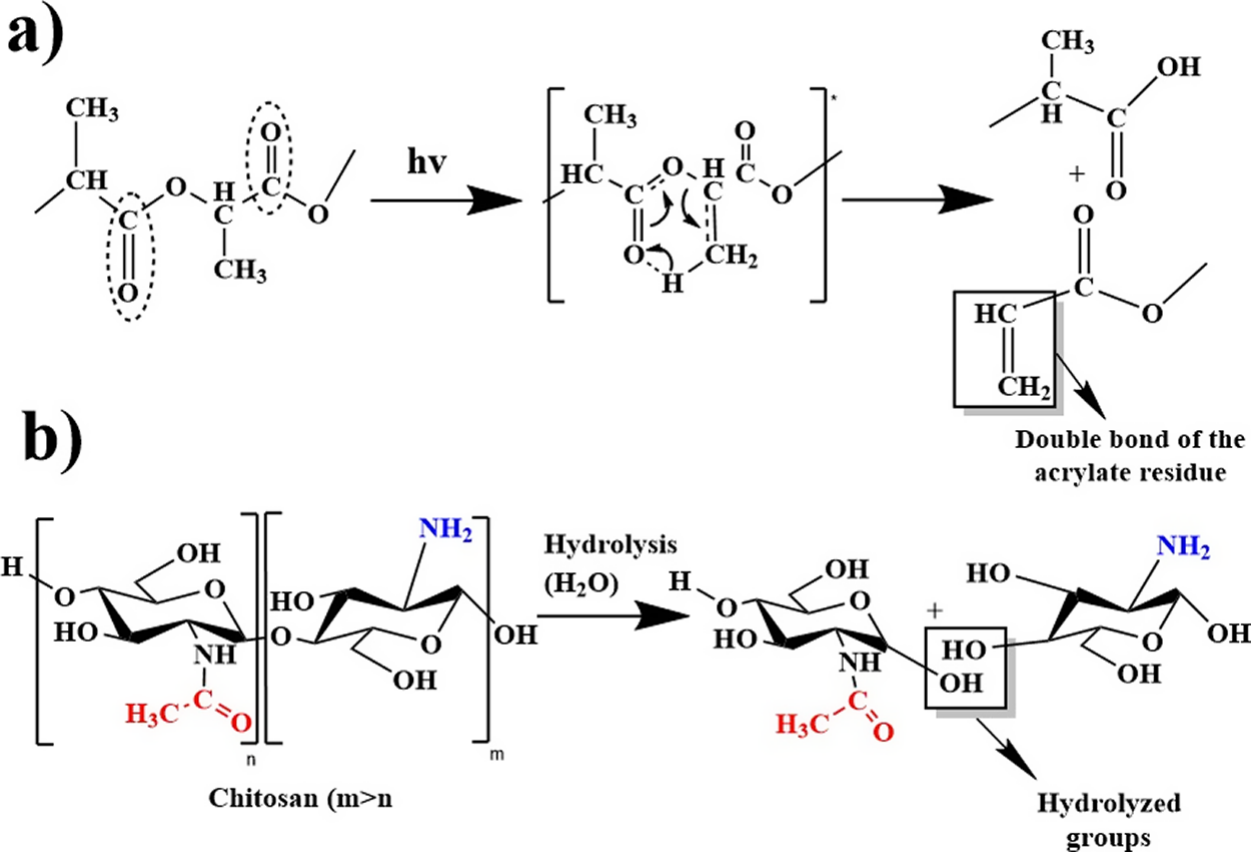
Proposed degradation mechanism for the films: (a) photodegradation of PLA via Norrish II and (b) hydrolysis degradation of chitosan.

Figure 4 shows the infrared spectra corresponding to the PLA films Q1 and Q2 exposed to different degradation times. The spectrum of the PLA film (Figure 4b) presented a new peak at around 895 cm^–1^ after 240 h of exposure. This signal is attributed to the symmetric bending of a double bond in acrylate residues resulting from the Norrish type II photodegradation of the polymer (Figure 5a).^23,24^ On the contrary, it proved challenging to identify other changes in the infrared spectrum associated with alternative degradation mechanisms, possibly due to this film’s pronounced resistance to degradation. On the other hand, the Q1 film (Figure 4c) displayed a new peak around 3412 cm^–1^ at 240 h of exposure, which can be attributed to the stretching of the hydroxyl (OH) group, associated with chitosan hydrolysis within the film (Figure 5b).^25^ As observed, this peak showed both a shift to a higher wavenumber and an increase in intensity during the final degradation time (T6), indicating a higher degree of hydrolysis and, consequently, a greater degradation of the film.^26^ Additionally, similar to the PLA, this film presented a peak around 895 cm^–1^ at 144 and 192 h of exposure (Figure 4d). Furthermore, during the latter two times T5-T6, another new signal was observed at 920 cm^–1^, which is attributed to the asymmetric bending of double bonds, as previously mentioned, is associated with the photodegradation of the PLA within the composite film.^24−27^ Finally, similar to Q1, the Q2 film exhibited the peak associated with hydrolysis at 3390 cm^–1^; however, this peak only appeared after 288 h of degradation (Figure 4e). Its appearance during the final degradation period (T6) may indicate the higher resistance of this film to the degradation process.

**Figure 6:**
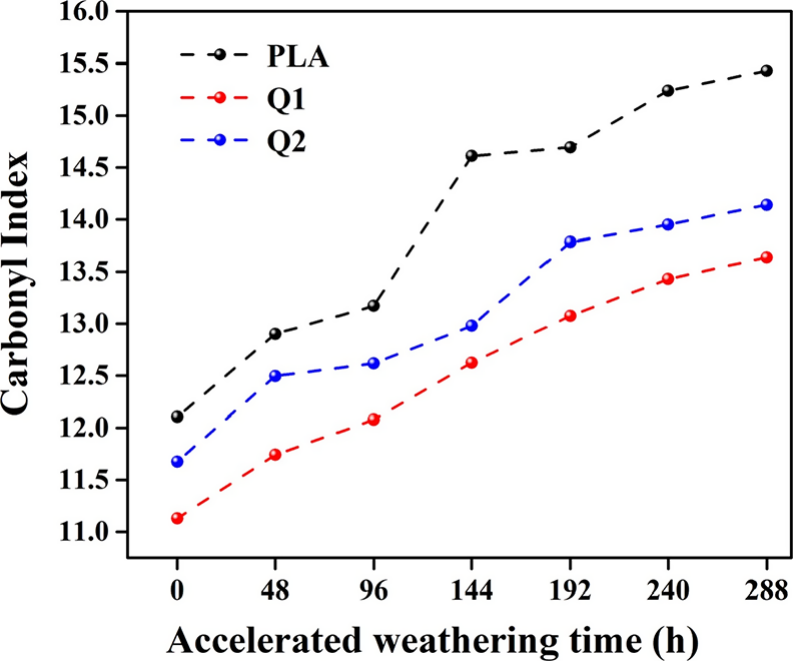
Carbonyl index of films at different exposure times.

At present, the carbonyl index is the most commonly employed indicator for monitoring the chemical oxidation and degradation of polymers. This choice is substantiated by reports indicating that when polymers are exposed to specific energy source, such as ultraviolet radiation, they can experience photooxidation reactions and subsequent chain breaks.^25−28^ Based on the above, the carbonyl index of the analyzed films was determined by calculating the ratio of the integrated absorbance of the carbonyl group band at 1750 cm^–1^ to that of a reference band corresponding to the symmetrical bending of the methyl group at 1453 cm^–1^, which remains insensitive to photooxidation.^29^Figure 6 illustrates the carbonyl index for the composite films exposed to accelerated degradation. An increase in the carbonyl index was noted for all films as the degradation time was extended, with the highest values recorded at 288 h of exposure. These results confirm the effective photodegradation for each composite film due to the effect of ultraviolet radiation.^30^

#### 3.2.2 Scanning Electron Microscopy

**Figure 7:**
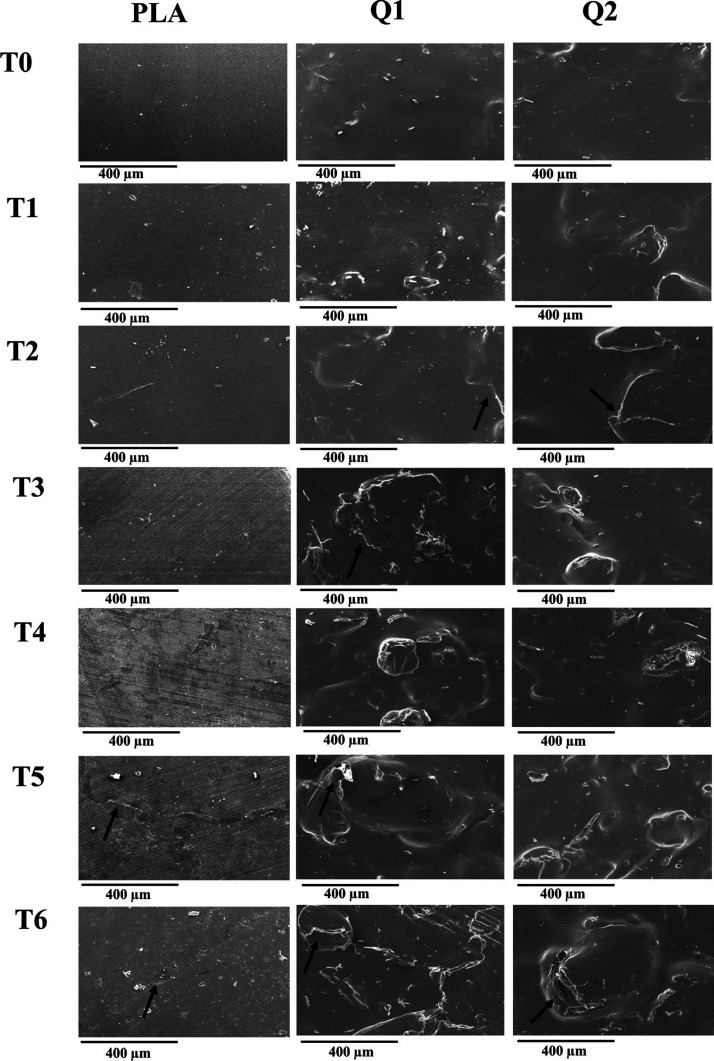
Micrographs of the surface of extruded films subjected to different exposure times of accelerated degradation. 150× magnification, scale bar 400 μm.

Using SEM, we conducted surface morphology analysis of the films before and after the accelerated degradation process (Figure 7). Prior to exposure to accelerated degradation (T0), the PLA film exhibited a smooth and homogeneous surface. However, upon the introduction of chitosan into the film (Q1), a loss of homogeneity was observed as chitosan displayed a tendency to form random agglomerates within the system. In contrast, the Q2 film, with MA, displayed improved homogeneity compared to the film without this component, resulting in the reduction of chitosan agglomerates within the PLA matrix. These findings suggest a compatibilizing effect of maleic anhydride in the composite film.^31,32^ Furthermore, we observed distinct changes in the morphology of the films during the degradation period. The PLA film exhibited alterations in its surface morphology starting at 192 h (T4), characterized by small isolated cracks. However, at 240 and 288 h (T5 and T6) of exposure, more substantial cracks became evident in specific regions of the material. On the other hand, both the Q1 and Q2 films exhibited crack formation as early as 96 h. Notably, the Q1 film displayed more extensive degradation compared with the Q2 film, showing larger cracks at each degradation time, primarily within the chitosan agglomerates. This observation highlights that reduced chitosan agglomerate formation in the PLA matrix, facilitated by the compatibilizing agent (MA), effectively mitigated material degradation under the given conditions. It is worth emphasizing that the most pronounced degradation effect was consistently observed at the final exposure time (T6) in both films.^33,34^

#### 3.2.3 Residual Mechanical Properties

**Figure 8:**
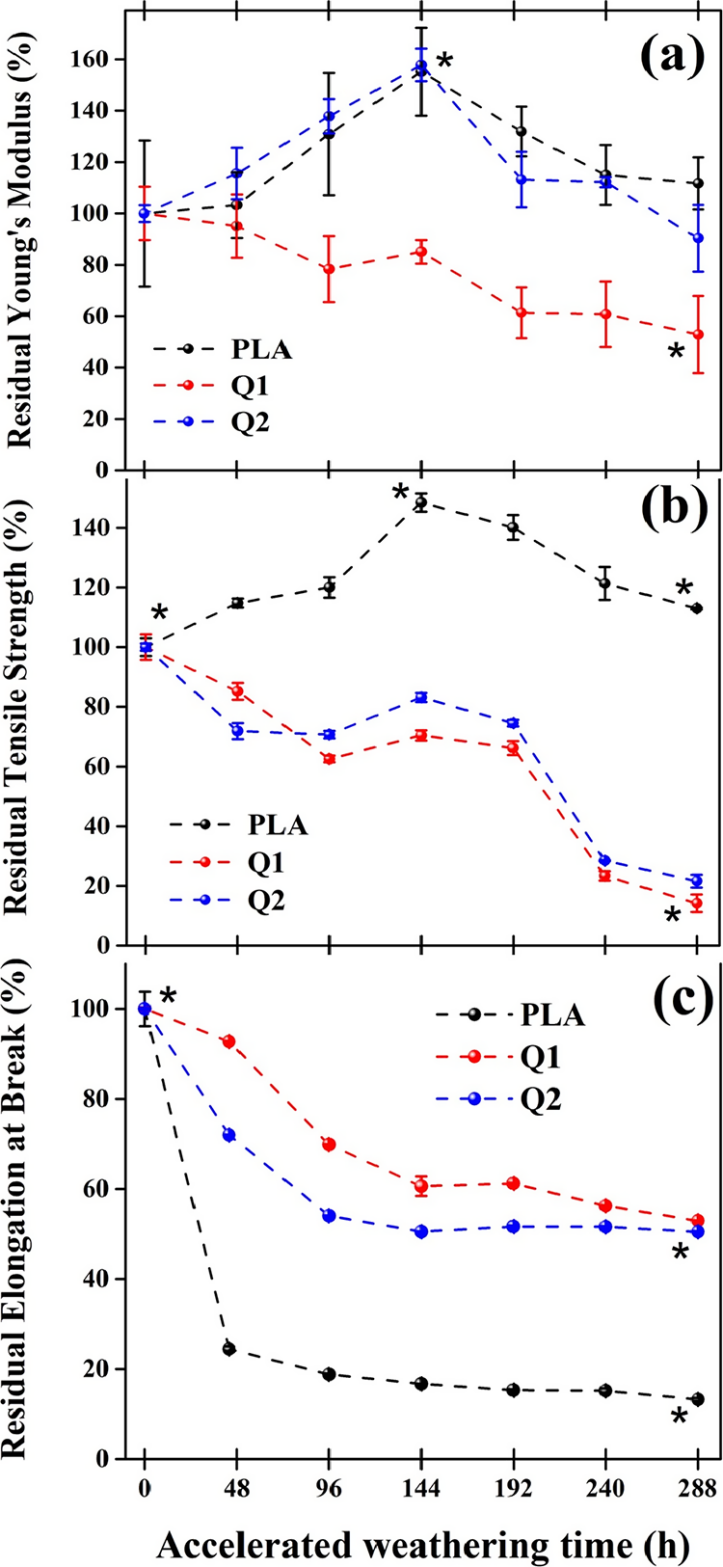
Residual mechanical properties of the films at different exposure times of accelerated degradation. (a) Residual Young′s modulus; (b) residual tensile strength; and (c) residual elongation at break. Asterisks indicate significant differences (*P* < 0.05).

In Figure 8a, the behavior of the residual elastic modulus (residual Young′s modulus) of the films at different exposure times was observed. The PLA film exhibited a gradual and significant increase in this property during the first 144 h, reaching a residual elastic modulus of 155.17%. This behavior can be attributed to cross-linking of the PLA chains due to the effect of ultraviolet radiation, where the formation of new covalent bonds reduces the mobility and flexibility of polymer chains, ultimately increasing the film′s rigidity.^35^ After 144 h of exposure, a decrease in the elastic modulus was observed. However, it is crucial to highlight that these values turned out to be higher than those of pristine PLA (T0). Specifically, in the last degradation period (288 h), the film exhibited a residual elastic modulus of 111.70%.

Similar to PLA, the Q2 film exhibited consistent behavior, reaching a maximum elastic modulus value of 157.81% at 144 h of exposure and a value of 90.37% at 288 h of exposure. This behavior can be attributed to the homogeneous distribution of chitosan in the PLA matrix, allowing for partial preservation of PLA properties. Q1 film, on the other hand, displayed a significantly different behavior compared to PLA and Q2 films. It exhibited a gradual decrease in the residual elastic modulus from the initial 48 h of exposure, maintaining this trend until the final degradation period (288 h), where it reached a residual modulus of 52.23%. This behavior could be attributed to the accumulation of chitosan at the interface of the polymeric matrix, leading to a reduction in cross-linking between polymer chains and, simultaneously, promoting the narrowing and rupture of the film.

The residual tensile strength analysis is shown in Figure 8b. The PLA film exhibited a gradual increase during the initial 144 h of exposure, reaching a value of 148.43%. This behavior is attributed to the cross-linking in the material, as mentioned earlier. Subsequently, a gradual decrease was observed, presenting a value of 113.45% at 288 h. However, even at this extended exposure time, the strength remains higher compared to the pristine PLA film (T0), suggesting a certain resistance to the degradation process of this film.^36^ On the other hand, the Q1 and Q2 films exhibited a continuous decrease in their residual tensile strength from the initial 48 h of exposure. Specifically, both films demonstrated a reduction of over 50% at 240 h, reaching final values of 14.16% for Q1 and 21.58% for Q2 at the end of the degradation period (288 h). When relating the observed behavior in the residual strength analysis to the results obtained through SEM (Figure 7), it is observed that the lower residual strength of the Q1 film is due to a higher presence of cracks associated with chitosan agglomerates within the film. This results in a reduced ability to withstand loads before crack propagation and film rupture. In contrast, the higher residual strength of the Q2 film may be associated with a lower formation of chitosan agglomerates, and consequently, fewer cracks within the film.^37^

In Figure 8c, the residual elongation at fracture is depicted, showing a consistent decrease in this property for all of the films over the course of exposure. Notably, the PLA film exhibited an initial value of 24.42% at 48 h, followed by a gradual decrease, ultimately reaching a residual elongation of 13.80% at 288 h of exposure. This trend aligns with the results obtained for the residual elastic modulus, indicating an increase in the film’s rigidity. In contrast, both the Q1 and Q2 films also experienced decreases in their elongation percentages, albeit to a lesser extent when compared to the PLA film. They exhibited values of 52 and 50.25%, respectively, at 288 h of exposure. Overall, the Q2 film displayed lower residual elongation in comparison to the Q1 film, which is consistent with the elastic modulus values observed in both films.

#### 3.2.4 Thermal Analysis

**Figure 9:**
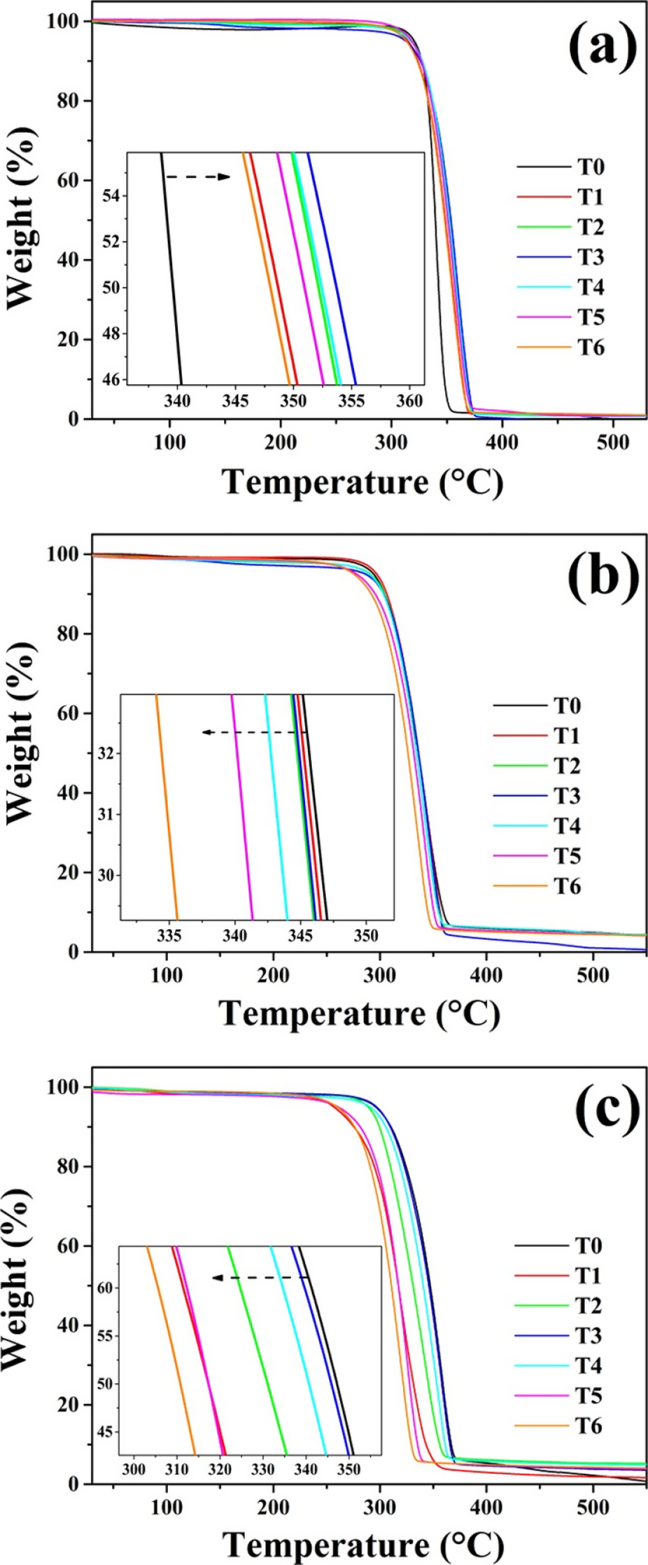
TGA thermograms of the films of (a) PLA, (b) Q1, and (c) Q2 at different exposure times of accelerated degradation.

The effect of accelerated degradation time on the thermal stability of the films was investigated by using TGA (Figure 9). In Figure 9a, the thermograms of the PLA films corresponding to each exposure period (T0–T6) are presented. A gradual increase in the inflection temperature was observed during the initial 144 h, reaching a maximum of 360.97 °C. This increase in thermal stability is associated with the enhanced formation of cross-links in the polymeric chains, aligning with the trends observed in the mechanical properties analysis (Figure 8).^35^ After 144 h (T3) of exposure, a shift in the inflection temperature trend was observed, with a gradual decrease over time, ultimately reaching a temperature of 352.14 °C after 288 h (T6). This behavior could be linked to the breakage of PLA chains, as proposed in the carbonyl index studies (Figure 6).^38,39^ On the other hand, the thermogram corresponding to the Q1 film is presented in Figure 9b. In contrast to the pure PLA film, the Q1 film experienced a decrease in its thermal stability from the first 48 h of exposure, a trend that continued until 288 h (T6), where it exhibited the lowest inflection temperature with a value of 336.26 °C. These results suggest a reduced resistance to degradation in the Q1 film compared to that in PLA, which could be related to the high susceptibility of chitosan to decompose under accelerated degradation conditions.^16,40^ Finally, the Q2 film exhibited a lower thermal stability at each of the exposure times compared to the pristine film (Figure 9c). However, some intriguing trends were observed. Specifically, the film displayed an inflection temperature of 341.90 °C at 48 h, which gradually increased to reach its highest value of 352.14 °C at 144 h. This behavior could be associated with the formation of cross-links in the polymeric chains, facilitated by the presence of MA. As the exposure time to radiation increases, MA has the ability to generate radical species that promote the formation of covalent bonds within the film.^41^ However, after 144 h, a decrease in the inflection temperature was observed, reaching a value of 333.84 °C at 288 h, which is related to the degradation of the material due to its exposure in the weathering chamber.

**Figure 10:**
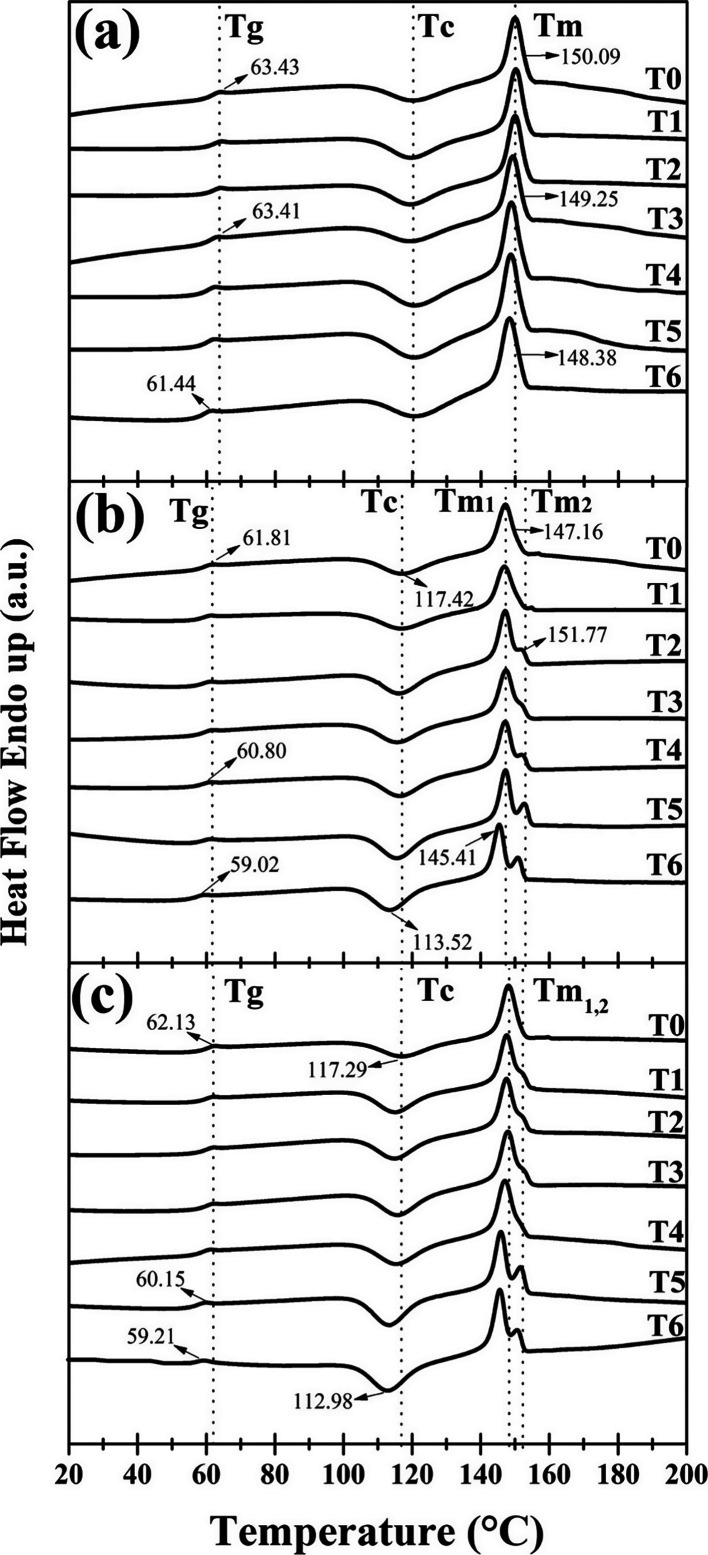
DSC thermograms of (a) PLA, (b) Q1, and (c) Q2 films at different exposure times of accelerated degradation.

The analysis by DSC of the composite films for each degradation period is depicted in Figure 10. As observed, the glass transition temperature (*T*_g_) and melting temperature (*T*_m_) for the pure PLA film (Figure 10a) remained constant during the first 144 h of degradation. Following this period, the film underwent a gradual decrease, reaching values of 61.44 and 148.38 °C for *T*_g_ and *T*_m_, respectively, at 288 h of degradation (T6). This decline could be associated with the photolysis of PLA chains, as observed in the results obtained from FTIR and carbonyl index analyses.^39,42^

On the other hand, the nondegraded Q1 film (T0) exhibited a *T*_g_ of 61.81 °C, which remained constant between times T1–T5 (Figure 10b). However, at 288 h (T6) of exposure, corresponding to the last degradation period, a shift to a lower temperature was observed, showing a corresponding *T*_g_ value of 59.02 °C. As mentioned earlier, this behavior could be associated with the increased mobility of the polymeric chains, mainly due to the degradation of chitosan in the composite film.^19^ Additionally, a decrease in the crystallization temperature (*T*_c_) of the film was observed after the first 48 h, maintaining this trend throughout the rest of the accelerated weathering process (T1–T6). This can be attributed to the rearrangement of the polymeric chains favored by the degradative process under accelerated weathering conditions.

The Q1 film also displayed significant changes in crystallinity percentages (*X*_c_ %). The pristine material (T0) had a crystallinity of 39.20% (Table 3), which gradually increased with exposure time, reaching a maximum increase of approximately 6% at 288 h. This behavior is related to the reorganization or migration of degraded amorphous segments into crystalline phases, known as chemo-crystallization and has been reported by various researchers.^43,44^ Conversely, the transition assigned to the melting of the material (*T*_m1_) occurred at 147.16 °C at T0. Furthermore, at 96 h (T2), the appearance of a second endothermic peak (*T*_m2_) at 151.77 °C was observed, gradually increasing in intensity with the exposure time. This could be correlated with the decrease in the crystallization temperature of the film. When crystallization occurs at a lower temperature, fewer perfect crystals are formed, and these can recrystallize at higher temperatures. Thus, the formed crystals (recrystallized) are more stable and contribute to the second *T*_m2_ transition.^45^ Additionally, a gradual decrease in the *T*_m1_ values was observed with respect to the exposure time, showing a value of 145.41 °C at 288 h. This behavior may indicate a decrease in the molecular weight of the material due to the degradation process. Finally, as shown in Figure 10c, the Q2 film exhibited slight shifts in *T*_g_ in the last two degradation times T5–T6. Additionally, similar to the Q1 film, a decrease in the crystallization temperature and a gradual increase in the crystallinity percentages over degradation time were observed, indicating effective degradation of this film.

**Table 3:** Crystallinity Percentages of Films Subjected to Different Exposure Times of Accelerated Degradation

accelerated weathering time (h)	*X*_c_ (%)			Δ*H*_cc_ (J/g)			Δ*H*_m_ (J/g)		
	PLA	Q1	Q2	PLA	Q1	Q2	PLA	Q1	Q2
0	36.81	39.20	33.90	–13.60	–17.98	–12.89	20.89	18.75	18.84
48	38.14	41.45	39.26	–15.39	–16.98	–15.93	20.34	21.85	20.86
96	38.30	42.31	40.45	–16.56	–18.32	–15.16	19.32	21.32	22.74
144	39.64	43.94	41.57	–18.19	–18.23	–19.02	18.95	22.94	19.95
192	39.90	44.62	41.60	–16.72	–20.13	–17.94	20.63	21.6	21.04
240	39.74	44.33	41.25	–15.90	–17.96	–16.66	21.33	23.31	21.99
288	39.79	45.41	43.26	–13.95	–17.43	–16.17	23.33	25.11	24.37

## 4 Conclusions

In conclusion, the successful production of PLA/chitosan films, with and without MA, was achieved through the extrusion molding technique. The presence of chitosan in Q1 films favored the degradative process, evident through crack formation, especially after the initial 96 h (T3) of degradation. Conversely, the addition of MA mitigated this process in the Q2 film, attributed to the reduction of chitosan clusters through compatibilization. All films exhibited an increase in the carbonyl index over the accelerated weathering time, indicative of a photodegradation process. However, the Q1 and Q2 films were notably more affected by humidity conditions, as evidenced by the increased intensity of the hydroxyl group in the infrared spectrum. The incorporation of chitosan significantly affected the mechanical properties, leading to a reduction in tensile strength of over 50% after 244 h of exposure. Nevertheless, the films with MA showed higher values in tensile strength and elastic modulus compared with those without MA (Q1), confirming greater resistance to degradation of the Q2 films. Regarding thermal stability, pure PLA films exhibited an increase in their inflection temperature during the initial 144 h, associated with the cross-linking of their chains due to the effect of UV radiation. In contrast, the Q1 and Q2 films decreased their thermal stability due to the scission of polymeric chains. To summarize, the addition of chitosan significantly influenced the physicochemical and mechanical properties of the composite material during accelerated degradation. These findings provide a valuable foundation for future research, particularly in the development of disposable food packaging.
